# Malaria control in Botswana, 2008–2012: the path towards elimination

**DOI:** 10.1186/1475-2875-12-458

**Published:** 2013-12-20

**Authors:** Chihanga Simon, Kentse Moakofhi, Tjantilili Mosweunyane, Haruna Baba Jibril, Bornapate Nkomo, Mpho Motlaleng, Davies Sedisa Ntebela, Emmanuel Chanda, Ubydul Haque

**Affiliations:** 1 National Malaria Programme, Ministry of Health, Gaborone, Botswana; 2 World Health Organization, Botswana Country office, Gaborone, Botswana; 3 Population Services International, Juba, Republic of South Sudan; 4 W. Harry Feinstone Department of Molecular Microbiology & Immunology, Bloomberg School of Public Health, Johns Hopkins University, Baltimore, MD, USA

## Abstract

**Background:**

Botswana has made substantial progress towards malaria elimination across the country. This work assessed interventions and epidemiological characteristics of malaria in Botswana, during a period of decreasing transmission intensity.

**Methods:**

National passive malaria surveillance data for five years (2008–2012) were analysed. A district-level, random effects model with Poisson regression was used to explore the association between malaria cases and coverage with long-lasting insecticide-treated nets (LLINs) and indoor residual spraying (IRS). Malaria cases were mapped to visualize spatio-temporal variation in malaria for each year.

**Results:**

Within five years, a reduction in malaria prevalence (approximately 98%) and number of deaths (12 to three) was observed. Between 2008 and 2012, 237,050 LLINs were distributed and 596,979 rooms were sprayed with insecticides. Coverage with LLINs and IRS was not uniformly distributed over the study period and only targeted the northern districts with a high malaria burden. The coverage of IRS was associated with a reduction in malaria cases.

**Conclusions:**

Botswana has made significant strides towards its goal of country-wide elimination of malaria. A major challenge in the future will be prevention and management of imported malaria infections from neighbouring countries. In order to accurately monitor progress towards the elimination goal, the malaria control programme (NMP) should strengthen the reporting and capturing of data at household and individual level. Systematic, periodic operational research to feedback the NMP will help to guide and achieve elimination.

## Background

Malaria is a major global health problem and a leading cause of morbidity and mortality. In 2010 the disease caused an estimated 219 million cases and 660,000 deaths with almost 80% of the cases and 90% of the deaths reported from Africa [1]. The World Health Assembly and Roll Back Malaria (RBM) targets for malaria control and elimination aim to achieve at least a 75% reduction in malaria incidence and deaths by 2015 [1]. To achieve the objectives of malaria control and elimination programmes, endemic countries are deploying a combination of established and newly-available highly effective malaria interventions, including: diagnosis and treatment of confirmed cases with artemisinin-based combination therapy (ACT), vector control interventions using long-lasting insecticide-treated nets (LLINs) coverage targeted to reach 90% of households’ and indoor residual spraying (IRS) coverage targeted to reach more than 80% house structures supplemented with larval source management [1].

Malaria control activities in Botswana started in the 1950s with a programme that focused mainly on vector control using IRS with diethyl-dichloro-trichloroethane (DDT) [2]. In the past decade, the country has made great strides towards implementation of key, high-impact interventions recommended for malaria control in accordance with international calls. Passive surveillance through monthly reports by integrated disease surveillance and response (IDSR) has been the mainstay of surveillance. Following the comprehensive national malaria programme (NMP) review of 2009, Botswana adopted the move towards malaria elimination and the target was set for 2015 [2]. This process also implemented strengthened vector control by scaling-up other interventions, such as mass distribution of LLINs and introduction of winter bio-larviciding. IRS has remains the main stay of malaria vector control as outlined in the 2010–2015 national malaria control strategic plan [2].

Malaria elimination became a reality in Botswana because of a significant reduction in malaria cases over the past few years [1]. Malaria is no longer a major public health problem and Botswana is implementing malaria elimination strategy. As the country moves forward with the malaria elimination efforts, in order to guide and refine control interventions it is crucial to monitor the trends in morbidity and mortality due to malaria in the affected populations. High coverage of interventions must become more geographically targeted. The current paper describes trends in malaria morbidity and mortality, improved diagnosis system, IRS and LLIN coverage, and association of IRS, LLIN and reported malaria cases using passive surveillance data routinely collected by the NMP, Botswana from 2008 to 2012.

## Methods

### Study area

**Figure 1:**
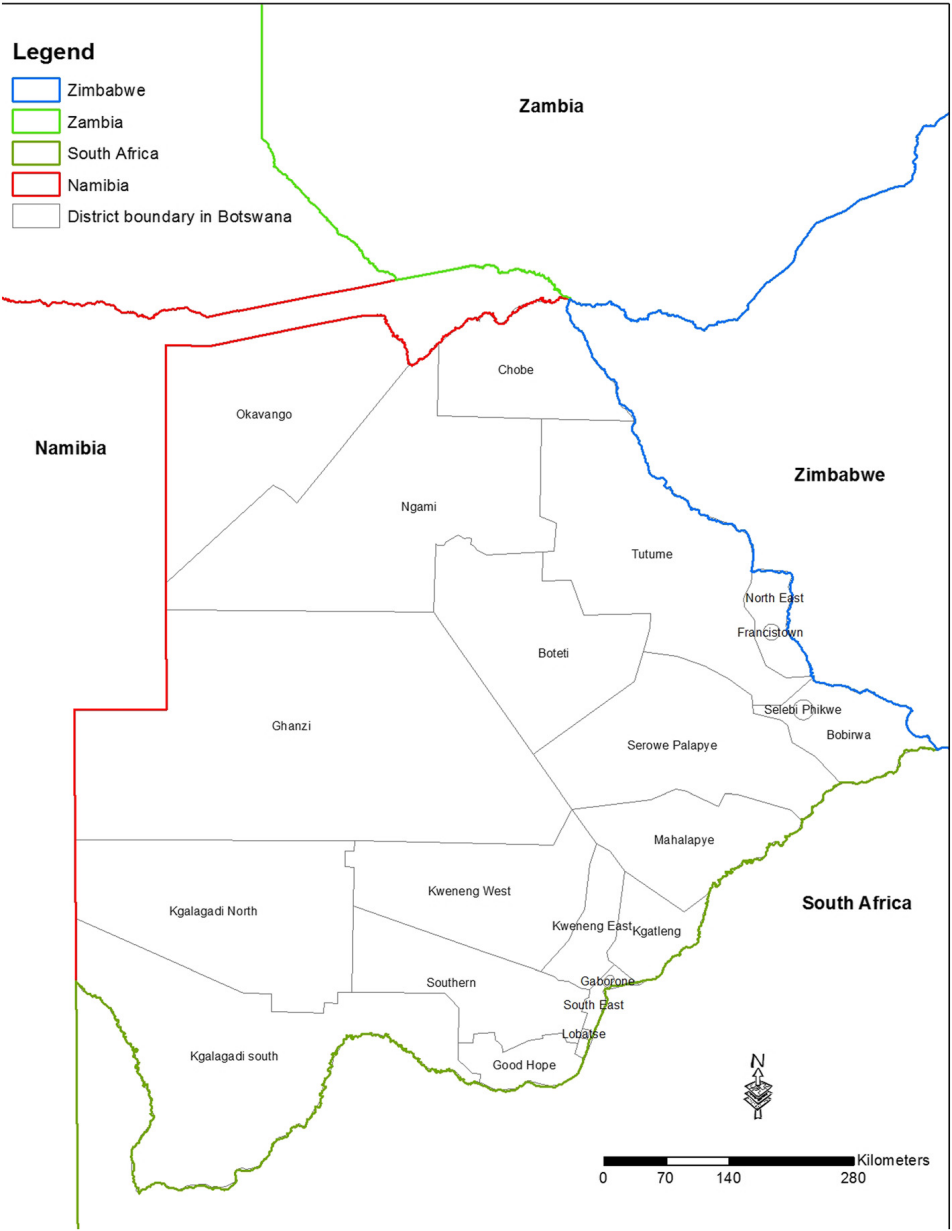
Map of Botswana.

Botswana is a semi-arid, land-locked country with a surface area of approximately 582,000 sq km. It shares borders with Zambia in the north, Namibia in the west, Zimbabwe in the east and South Africa in the south (Figure 1). The average daily temperature ranges from 22 to 33°C in January. The daily minimum temperature ranges from 19°C in January to -5°C in July. The rainy season is from October to April, with the average annual rainfall ranging from 250 mm in the southwest to 650 mm in the northwest [2,3]. Malaria transmission in Botswana is seasonal and unstable with some recorded sporadic outbreaks [2]. Transmission levels differ from year to year depending on both rainfall and temperature [2]. The country has progressed through various stages of malaria control (Table 1).

**Table 1:** Chronology of key milestones for malaria control in Botswana

**Year**	**Key milestones**
1950	Malaria control activities in Botswana started in 1950s with a programme that focused mainly on vector control using IRS with DDT
1974	A comprehensive programme was initiated with emphasis on a number of components (vector control, case management and health education)
1996	Malaria vector control through IRS as a vertical programme was decentralized from central government to district level under the primary health care approach
1996	Weekly notification of malaria cases introduced with indicators of confirmed, unconfirmed and malaria deaths being reported
1997	NMP Botswana introduced the use of insecticide-treated nets (ITNs) as a complementary strategy to IRS.
1998	Sulfadoxine–pyrimethamine introduced as first-line treatment following evidence of resistance to chloroquine between 1994 and 1997
2003	IDSR strategy introduced incorporating malaria indicators
2007	NMP introduced ACT adopting artemether-lumefantrine as first-line treatment for uncomplicated malaria and RDT for malaria diagnosis in all districts
2009	In line with the move to malaria elimination, a policy change required all cases to be tested before treatment
	Re-introduction of DDT for IRS and switch to use of ITNs to LLINs as strategy to achieve elimination
	Following a comprehensive NMP review, Botswana adopted move towards malaria elimination, and the target was set for 2015
2010	Malaria policy developed to guide implementation of malaria interventions. Malaria Strategic Plan 2010 –15 using recommendations from programme review of 2009.
	Mass free distribution of LLINs adopted
2012	Case-based surveillance introduced

### Malaria morbidity and mortality by district

Annually aggregated, district-level malaria case data were obtained from the Botswana NMP. Malaria indicators collected and reported by the programme are clinical cases, confirmed malaria cases and deaths disaggregated by age, sex, and inpatient and outpatient status. Clinicians use tally sheets at health facility level. Data from consultation rooms is consolidated both weekly and monthly at health facility level into a report which is submitted to districts and national level each month. Diseases targeted for elimination like malaria are reported immediately to the district and national level through quickest means possible and notified through a disease specific notification form. Malaria death investigation forms were completed by attending medical officers and were submitted to the NMP for analysis. There were no community deaths, so no verbal autopsies were done.

### Population data

The country had a relatively small population of approximately 2.04 million in 2011 [4]. Botswana’s population was projected in each district for 2008, 2009, 2010 and 2012 using an exponential population growth model based on 2001 and 2011 Census [4,5].

### IRS and LLIN coverage by district

A data collection tool was used to enter the number of rooms sprayed by IRS in each district. These figures were then submitted to district supervisors who in turn produced districts reports that were later consolidated at national level for each year. The number of population in each district was projected for 2008, 2009, 2010 and 2012 based on 2001 and 2011 estimates [4,5], using an exponential growth model. The IRS coverage rates were calculated as the number of rooms sprayed in each house/number of rooms found. NMP only targeted living rooms for spraying.

The NMP distributed LLINs door-to-door based on sleeping spaces or one net for every two people in cases where sleeping spaces are not defined. They demonstrated to household members how to hang nets and provided education on how nets work. Before and during the malaria transmission season there were campaigns to encourage people to hang their nets (“Keep Up” campaigns). The LLINs were also distributed to children under the age of five through child welfare clinics and to pregnant women through antenatal clinics. Assuming an average net life of three years, district-level coverage rates of LLINs were calculated per person [6]. LLIN rates in each district were calculated as the number of nets per two household residents using the projected population denominator for each district.

### Larviciding at pilot stages

Biolarviciding with *Bacillus thuringensis* var*. israelensis* (Bti), Bugstop®, manufactured by Regent Laboratories, South Africa was implemented in Bobirwa district in 2012. During the winter season, district malaria vector control teams with technical support from the Entomology unit of the Ministry of Health identified water bodies within 2 km of human settlements. Prior to larvicide application, baseline data on larval densities, location and size of water bodies was collected and mapped using global positioning system. Granular Bti was applied at a dosage of 2.0 g/sq m of water using two types of technique: 1) a hand spreader for application in smaller water bodies, and 2) hand broadcasting for relatively larger water bodies. The first larvicide treatment was done in August/September 2012 and the second in October 2012. Larval densities were determined by making several scoops of 354 ml-capacity ladle and then averaging the number of larvae per scoop. Monitoring was done before treatment, at 24 hr, 48 hr and at three-week intervals post-treatment at Mathathane reference point.

### Statistical analysis

Using projected demographic data as denominator, aggregated annual data for IRS, LLINs and malaria prevalence (number of events in a given population at a designated time [7]) were analysed for all districts in Botswana. A district level random effect model was used in Poisson regression analysis here to explore the association between malaria rates and LLIN and IRS coverage at the district level. To ensure that standard errors incorporated the effects of over-dispersion, we computed robust standard errors that bring in any extra-Poisson variability [8]. STATA 11 (STATA Corp 2003, College Station, TX, USA) was harnessed for all statistical analyses.

### Hotspot analysis

The district shape files (geographic data) obtained from the Ministry of Health were linked with malaria prevalence data. District-level annual malaria prevalence data were mapped and analysed independently for spatial clustering (or hotspots) using the Getis-Ord Gi* statistic [9] in the ArcGIS software (ESRI, Redlands, CA, USA). The z-scores and p-values, reflecting whether the differences between the local and global means are statistically significant [10] were produced for each district by comparing the local Getis-OrdGi* statistic local mean rate (the rates for a district and its nearest neighbouring districts) to the global mean rate (the rates for all districts). A statistically significant positive z-score indicates a hotspot for high rates. Similarly, a statistically significant negative z-score for a district indicates local spatial clustering of low rates [8,9,11,12].

## Results

### Changes in the burden of malaria

Malaria cases reduced from 17,886 (0.97% prevalence) in 2008 to 311 (0.01% prevalence) cases in 2012 (Table 2). Malaria-attributable deaths reduced from 12 in 2008 to three in 2012 (Table 2). Considering 2008 as baseline a smooth decline was observed until 2012. The prevalence of malaria in all districts in 2012 was less than 0.02% except in Bobirwa district where prevalence was 0.1%. No clear trend was reported about severe anaemia among individuals under five years until 2010 when a decline was reported. Only 63% of the reported cases were confirmed by either microscopy or rapid diagnostic test (RDT) in 2012 (Table 2).

**Table 2:** Reported malaria cases, diagnostic tests and interventions in Botswana, 2008 – 2012

**Year**	**2008**	**2009**	**2010**	**2011**	**2012**
Malaria prevalence (%)	0.97	0.80	0.62	0.08	0.01
% confirmed cases	7	6	9	30	63
% clinically diagnosed malaria cases	93	94	91	70	37
Severe anaemia <5 (number)	53	23	40	10	6
Number of malaria-related deaths	12	7	8	8	3
LLIN coverage per person	0.18	0.61	1.4	1.94	2.11
IRS coverage (% of rooms)	67	72	80	67	65

### Hotspots

**Figure 2:**
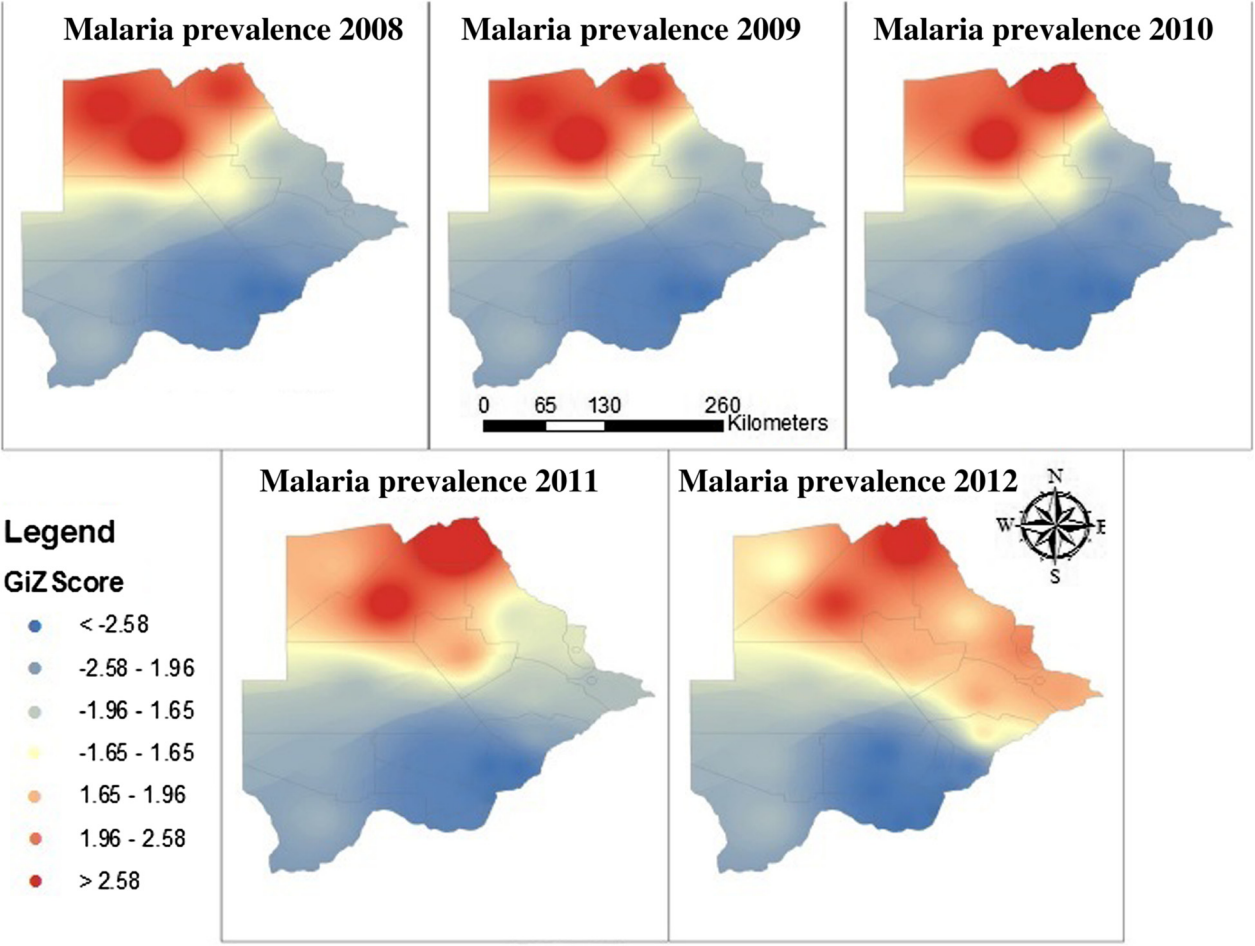
Malaria hotspots in Botswana.

Malaria transmission levels vary significantly within the country with more cases being reported in the northern part of the country (Figure 2). Significant malaria hotspots (p <0.01; z >2.58) were reported in only three districts (Chobe, Okavango and Ngami district) in northern Botswana from 2008 to 2010. Significant malaria hotspots were reported in only two districts (Chobe and Ngami district) in 2011. Okavango district accounted for 22% of Botswana’s total cases reported from 2009 to 2012. The remainder of the districts experienced sporadic malaria cases. Although malaria cases declined in 2012, with three districts reported zero cases, some districts (Kweneng East, West Kgatleng, Palapye, Mahalapye, and South East) reported malaria hotspots in southern and central Botswana. Malaria transmission in the country has decreased over the years, and became limited to the districts along the borders with Zambia, Zimbabwe and Namibia. Bobirwa district sharing border with South Africa continuously reported significant lower clustering of malaria cases.

### Vector control activities

**Figure 3:**
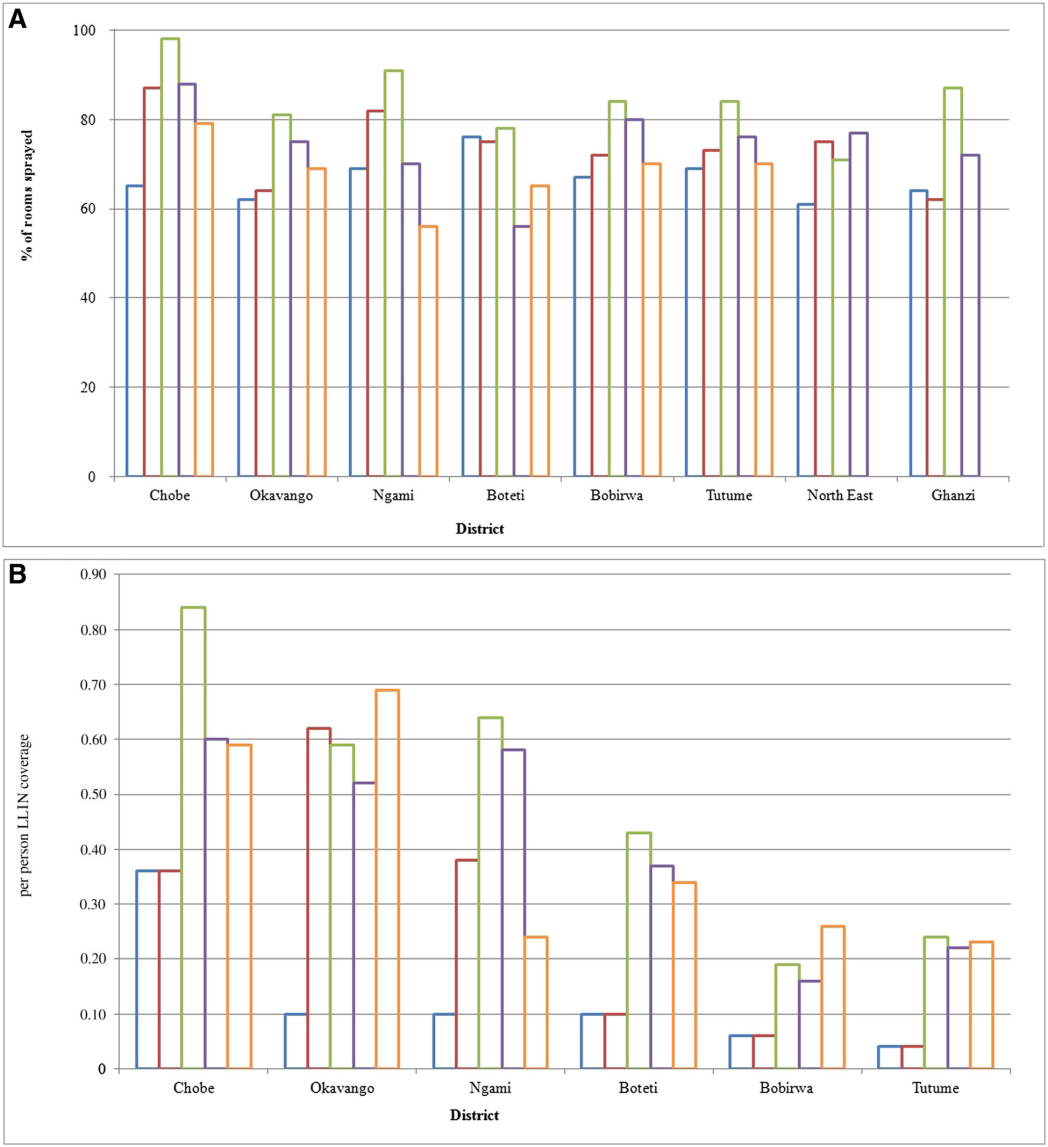
**IRS and LLIN coverage 2008-2012. (A)** Blue, red, green, violet and orange square symbol represents IRS coverage for 2008, 2009, 2010, 2011 and 2012 respectively. **(B)** Blue, red, green, violet and orange square symbol represents LLINs coverage for 2008, 2009, 2010, 2011 and 2012 respectively.

From 2008 to 2012, IRS was conducted in 596,979 rooms. The coverage of IRS ranged from approximately 56% (lowest) rooms in Ngami district to 79% (highest) rooms in Chobe district in 2012. The coverage of LLINs was restricted to six districts from 2008 to 2012. Over the same time period, 237,050 LLINs were distributed. In 2012, LLINs coverage was highest (0.69 LLIN per person) in Okavango district. Both IRS and LLINs coverage rate varied over the years (Figure 3).

### Associations between vector control activities and the burden of malaria

An additional increase in IRS coverage by 1% will reduce malaria by a factor of 0.75, while holding LLINs in the model constant. LLIN was not associated with reduced malaria cases (Table 3). It separately revealed the estimated rate ratio for one unit increase in IRS and LLINs standardized score, given the other variable is held constant in the model. If Botswana is to reduce malaria cases by 1%, the country needs to increase IRS coverage by a factor of 0.75, while holding LLINs in the model constant.

**Table 3:** Impact of indoor residual spraying and long-lasting insecticide-treated nets on malaria prevalence in Botswana, 2008 - 2012

	**Total prevalence**
	PRR* (95% CI)
**IRS**	0.75 (0.63-0.90)
**LLIN**	1.19 (1.09-1.28)
**Year**	
2008	1
2009	0.80 (0.77-0.82)
2010	0.64 (0.61-0.67)
2011	0.08 (0.07-0.08)
2012	0.01 (0.01-0.02)

*PRR Prevalence rate ratio.

### Larviciding at pilot stages

The 48-hr post-application monitoring at Mathathane treated site showed 60% larval reduction of anopheline larvae. Significant reductions in larval densities were observed 28 days after the first treatment with ≥89% anopheline larval reduction and ≥75% culicine larval reduction between August/September and October 2012 treatments.

## Discussion

Botswana made remarkable progress in reducing malaria cases between 2008 and 2012, in part through targeted coverage with vector control interventions. This reduction in the burden of malaria was associated with IRS coverage at the district level. These measures included re-introduction of DDT for intensified IRS, free mass distribution of LLINs, larviciding and intensified community mobilization campaigns to educate the public on IRS, LLINs and early treatment. These measures were publicized through electronic and print media [1]. Other contributing factors are training and retraining of health workers in malaria case management which has been intensified, has helped improve malaria diagnosis and treatment [1], improved access to health facilities, leading to early treatment as 95% households are within 8 km of health facilities [13] and treatment of all clinical cases with ACT, in line with national guidelines. The very recent use of confirmed diagnosis (in 2011 and 2012) coincides with the decreased reports of malaria cases so decreases may reflect improved diagnostic capabilities to identify cases.

Despite the significant decline in reported malaria cases in Botswana, persistent hotspots remained in Chobe, a district sharing a border with Zambia. Significant malaria hotspots were reported in 2012 in districts Bobirwa, Chobe and Tutume that share border with Zimbabwe. With only 313 malaria cases in 2012, there is need to map all positive households for more targeted interventions. To achieve the country’s malaria elimination goal, strong cross-border collaboration, such as the recent Trans-Zambezi Malaria Initiative between Botswana, Zambia, Zimbabwe, Namibia and Angola would be critical [14]. Regular border screening among travelers may contribute to the reduction in malaria transmission. Understanding the origin and pathways of malaria transmission into Botswana will facilitate the development of effective strategies to mitigate and manage imported malaria and the risk of future transmission.

Botswana conducted IRS and distributed LLINs in districts that reported high malaria cases and performed targeted interventions. The reasons for the changing pattern of malaria epidemiology and declining disease burden are most likely multifactorial. IRS coverage in parts of northern Botswana could have initially contributed to the fall of malaria transmission, although it is unlikely that the increase in malaria risk due to LLIN is causal, and the reason might be that more affected areas were given more nets. Winter biolarvicing may have contributed to reduce malaria cases in Bobirwa district. Since insecticide resistance is evident in 37 African countries [1] including low endemic settings like Zanzibar [15], mapping and monitoring of insecticide resistance to secure sustainable reduction in Botswana will help to achieve elimination.

Although IRS coverage contributed to the decline of malaria cases in Botswana, the main reasons for rapid decline remain unknown, as in other African countries [16-19]. To ensure sustainable and smooth elimination, improved recording system (by age, sex, locality, travel history among positive cases), seasonality and risk factor identification among positive houses will help the country achieve sustainable malaria control. Data collection should be well designed and rigorously undertaken, and a high standard of programme management is essential. Elimination needs a relentless focus on surveillance and response, including mass screening and treatment around 500 m from positive households [13], enhanced larviciding programmes, and identification and rapid elimination of foci of all infections, including both symptomatic and asymptomatic. Nationwide use of mobile telephone for malaria diagnosis and treatment can accelerate malaria elimination [20]. Operational research regarding malaria related treatment seeking behavior [21], and malaria risk mapping [22-24] in Botswana will be crucial for targeted interventions. Seasonal malaria can be targeted for intervention since rainfall and sea surface temperature (SST) have already proved to be predictors of malaria cases in Botswana [25]. Both rainfall and annual malaria anomalies in December–February were significantly correlated to SST in the eastern Pacific [26].

Botswana is targeting malaria elimination by 2015. The programme is in the process of re-orienting towards malaria elimination. Case based surveillance implementation started in October, 2012 following training of health workers from all districts. Surveillance has been strengthened and provision of vector control interventions in non spraying districts is determined now by entomological investigations. Programme is in process of identifying transmission foci and plans to target interventions. A midterm review of the strategic plan conducted in October 2013 indicated that Botswana is still within reach of its 2015 target, but there is need to strengthen surveillance and targeting of vector control interventions. Achieve and maintenance of malaria elimination in Botswana will be challenging [27]. Importation of malaria cases in Botswana from endemic countries can jeopardize elimination goal [28]. Major obstacles to malaria elimination in low endemic setting like Zanzibar including trained manpower, and inadequate malaria education can be a lesson for Botswana too [29]. There is inadequate funding for elimination as the programme is mainly funded by Botswana government with limited LLIN donation by ‘the UNICEF’, ‘Malaria No More’, ‘Government of Japan’, ‘Anglican Church’, ‘Red Cross’, ‘Rotary foundation’ and ‘Indian Government’. International donor funding will be helpful to support the elimination goal.

These analyses were based on routinely collected data within the IDSR. There was no independent verification. Measure was only numbers of LLINs distributed and rooms sprayed. The observed associations between LLIN and IRS coverage and the burden of malaria were ecologic and not at the level of individuals. Many cases were not confirmed by RDT and microscopy but were based on clinical signs and symptoms, with the potential for misclassification. Both RDT and microscopy have limited sensitivity and specificity and are particularly likely to misclassify individuals with low levels of parasitaemia. It is probably not all improvements in the malaria situation were due to the control activities of the NMP. Botswana has undergone significant progress in terms of general stability and economic development. Improved access to healthcare could have contributed in malaria reduction irrespective of the activities of the NMP. Factors unrelated to specific malaria interventions, including agriculture practices, economic development, housing construction, and environmental management of breeding sites for mosquitoes might have changed over the past five years and contributed to the reduced risk of infection in the communities.

## Conclusions

Malaria elimination in Botswana has realistic possibility. Continuous efforts are critical to maintain the gains that have already been achieved. Cross-border malaria control initiatives with neighbouring countries are crucial to reduce malaria in the region and the importation of malaria into Botswana. Operational research and documentation regarding vector distribution and insecticide resistance status must be conducted as a matter of urgency. The surveillance systems must be refined and all positive houses should be mapped and regularly updated for targeted interventions and to ensure the information required to inform an elimination agenda are routinely collected.

## Competing interests

The authors declare that they have no competing interests.

## Authors’ contributions

UH and CS conceived the study design; UH performed data analysis and drafted the manuscript; CS, KM, TM, HBJ, BNK, MM, and DSN contributed to writing the manuscript; EC contributed to the writing and critically reviewed the manuscript. All authors approved the final version of the manuscript.
